# Protocol for detecting introgressed archaic variants with SPrime

**DOI:** 10.1016/j.xpro.2021.100550

**Published:** 2021-05-19

**Authors:** Ying Zhou, Sharon R. Browning

**Affiliations:** 1 Public Health Sciences Division, Fred Hutchinson Cancer Research Center, Seattle, WA 98109, USA; 2 Department of Biostatistics, University of Washington, Seattle, WA 98115, USA

**Keywords:** Bioinformatics, Genetics

## Abstract

The SPrime program detects the variants in current-day populations that were introgressed from an archaic source in the past. It is optimized for detecting introgression from Neanderthals and Denisovans in modern humans. We provide a protocol for detecting Neanderthal and Denisovan introgression in 1000 Genomes Project data, specifically focusing on the CHB (Han Chinese in Beijing) population.

For complete details on the use and execution of this protocol, please refer to Browning et al. (2018).

## Highlights

• This protocol describes how to run and interpret the results of SPrime
• SPrime identifies variants that are introgressed from an archaic source population
• Scripts for comparing the SPrime results to archaic genomes are provided

## Graphical abstract

**Figure:**
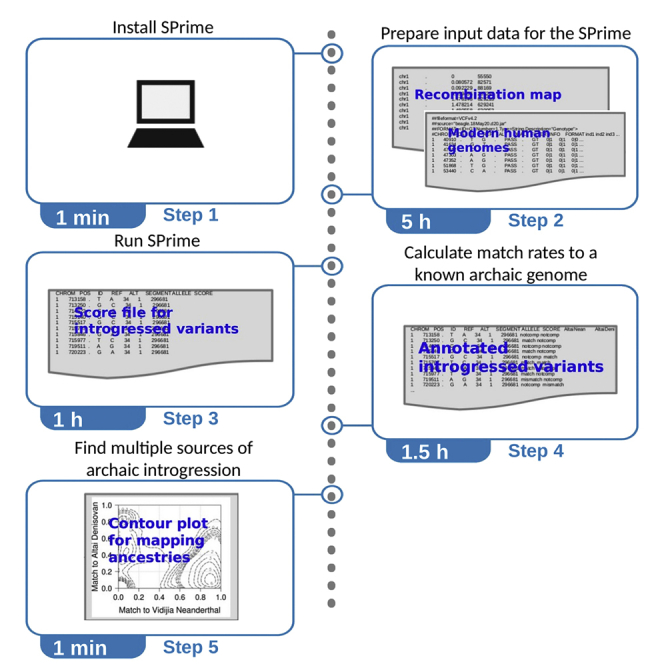

## Before you begin

### Download the script files

**Timing: 1 min**

1. The SPrime pipeline script files are available from github (see key resources table). In this protocol, we use different folders for the output from each step and for the source data. We use a folder named “download” to store all downloaded data, a folder named “tools” to store computation tools and scripts specific to this protocol, a folder named “tmp” to store temporary files, and folders named “step[2–5]” to store output from each step. The code for each step should be executed within the corresponding folder unless you modify the file paths in the code. Basic knowledge about bcftools, R scripting, and bash scripting is required to understand this protocol.

### Download the sequence data

**Timing: 5 h**

2. Sequence data for current-day and archaic populations should be in gzip-compressed VCF format (Danecek et al., 2011). The example data in this protocol can be downloaded via the links listed in the Key Resources Table. The script for downloading the genotype data from the 1000 Genomes Project, genotype data and genome masks for the two archaic populations, and a recombination map for the example analysis is available in the folder “download” in our published pipeline online (key resources table). In this protocol, we analyze the phase 3 data from the 1000 Genomes Project, as these are the data that were analyzed in the original SPrime paper (Browning et al., 2018). Analysis of the high-coverage 1000 Genomes data from the New York Genome Center's high coverage resequencing (Byrska-Bishop et al., 2021) would require the use of a genetic map in GRCh38 coordinates, and liftover of the SPrime results to match the GRCh37 coordinates of the available Neanderthal and Denisovan genomes.

## Key resources table

**Table:** 

REAGENT or RESOURCE	SOURCE	IDENTIFIER
**Deposited data**		

1000 Genomes Project data, phase 3 version 5a	The 1000 Genomes Project (1000 Genomes Project Consortium 2015)	ftp://ftp.1000genomes.ebi.ac.uk/vol1/ftp/release/20130502/
1000 Genomes Project data, high coverage	New York Genome Center (Byrska-Bishop et al. 2021)	http://ftp.1000genomes.ebi.ac.uk/vol1/ftp/data_collections/1000G_2504_high_coverage/working/20190425_NYGC_GATK/
Altai Denisovan and Vindija Neanderthal genomes, and genome masks	Kay Prüfer (Prufer et al., 2017)	http://cdna.eva.mpg.de/neandertal/Vindija/VCF/Vindija33.19/ http://cdna.eva.mpg.de/neandertal/Vindija/FilterBed/Vindija33.19/ http://cdna.eva.mpg.de/neandertal/Vindija/VCF/Denisova/ http://cdna.eva.mpg.de/neandertal/Vindija/FilterBed/Denisova/
HapMap genetic map	The International HapMap Consortium (The International HapMap Consortium 2007)	http://bochet.gcc.biostat.washington.edu/beagle/genetic_maps/

**Software and algorithms**		

SPrime	Brian Browning (Browning et al. 2018)	https://github.com/browning-lab/sprime
Bcftools	SAMtools	http://samtools.github.io/bcftools/bcftools.html
SPrime pipeline	This protocol	https://github.com/YingZhou001/sprimepipeline
R	R Project (R Core Team 2019)	https://www.r-project.org/

## Materials and equipment

• Genotype data (VCF files for current-day and archaic populations, see download the sequence data in before you begin)
• SPrime, bcftools, R and other scripts used in this protocol (see Software section of key resources table)
• A Linux computer with bash installed and at least 16 Gb of memory. The protocol should also work on Mac OS. In this protocol, all tests were run on a Linux 12-core 2.6 GHz computer with Intel Xeon ES-2630 processors and 128 GB of memory.

## Step-by-step method details

### Step-1: Install SPrime

**Timing: 1 min**

**Figure 1:**
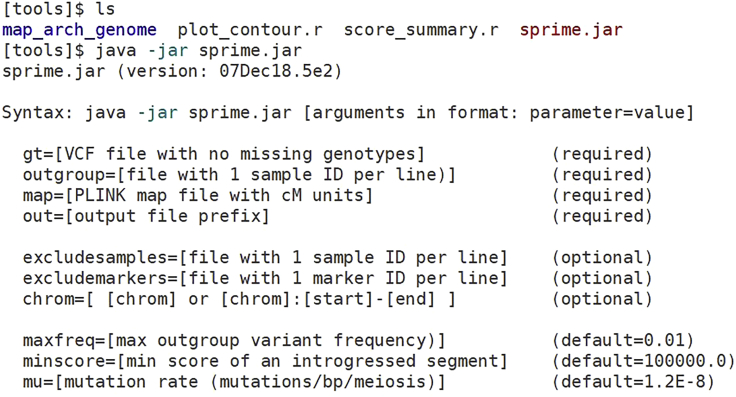
Screenshot showing the contents of the tools folder and sprime.jar’s help message

1. The SPrime software has been included in the SPrime pipeline download. The latest version of the software can be downloaded from the github page: https://github.com/browning-lab/sprime. Place the software file “sprime.jar” in the “tools” folder. Check that it is working and print out information on the parameters by running “java -jar sprime.jar” (Figure 1).

### Step-2: Prepare input data for the SPrime analysis

**Timing: 5 h**

2. SPrime takes the genotype data and recombination map as required inputs, along with a file specifying the outgroup samples. The example genotype data are downloaded from the 1000 Genomes Project. In this protocol we will use an East Asian population, the CHB (Han Chinese in Beijing, n=103), as the target group, and an African population, YRI (Yoruba in Ibadan, n=108), as the outgroup. We need to extract the samples of both the target group and the outgroup for each chromosome, and filter to remove all variants that are not bi-allelic SNPs as follows:
pfile=../download/1000genome/integrated_call_samples_v3.20130502.ALL.panel
grep -E "(YRI|CHB)" ${pfile} | cut -f1 > sample.txt
grep YRI ${pfile} | cut -f1 > outgroup.txt
echo -n "" > vcf.file.list
for chr in {1..22}; do
  vcf=../download/1000genome/ALL.chr${chr}.phase3_shapeit2_mvncall_integrated_v5a.20130502.genotypes.vcf.gz
  outvcf=../tmp/chr${chr}.vcf.gz
  echo ${outvcf} >> vcf.file.list
  bcftools view --samples-file sample.txt ${vcf} | bcftools view -c1 -m2 -M2 -v snps | bcftools annotate -x INFO,ˆFORMAT/GT -Oz > ${outvcf}
done

Extracting samples and filtering SNPs for all chromosomes takes about 5 h. The maximum memory in use is 13.5Mb.

3. We concatenate all autosomes into one file, because SPrime needs whole genome data to estimate key parameters. Although we will analyze the chromosomes one by one in order to parallelize computation, SPrime will obtain information about relative mutation rates from the whole autosome.
bcftools concat --file-list vcf.file.list --naive --output-type z --output all.auto.vcf.gz

### Step-3: Run SPrime to detect introgressed variants

**Timing: 1 h**

4. We use the HapMap combined LD map as the input recombination map in this example, following the analysis in (Browning et al., 2018). The recombination map must be in plink format (https://www.cog-genomics.org/plink/1.9/formats#map) and have the same genome build version and chromosome identifiers as the genotype data. Since the phase 3 1000 Genomes Project data uses GRCh37 coordinates, we use a build 37 map here.
map=../download/plinkmap/plink.all.GRCh37.map
5. SPrime requires specification of the genotype data “gt=[file]”, the outgroup sample list “outgroup=[file]”, the recombination map “map=[file]”, and the output prefix “out=[string]”. One can also specify the chromosome using “chrom=[chrom]”, or the target region using “chrom= [chrom]:[start]-[end]”. Here we parallelize the analysis by chromosome, so we use the “chrom=” parameter.
outgroup=../step2/outgroup.txt
gt=../step2/all.auto.vcf.gz
for chr in {1..22}; do
  map=../download/plinkmap/plink.all.GRCh37.map
  out=chb.yri.${chr}
  java -jar ../tools/sprime.jar gt=${gt} outgroup=${outgroup} map=${map} out=${out} chrom=${chr}
done

It takes approximately 1 h to run SPrime on all autosomes. The maximum memory in use for one chromosome is 4.6GB.

### Step-4: Calculate match rates to a known archaic genome

**Timing: 1.5 h**

SPrime is able to detect archaic introgression without knowing the archaic genome by utilizing a purported non-admixed population as an outgroup. For Neanderthal or Denisovan introgression, a West African population is typically used as the outgroup, for example the YRI from the 1000 Genomes Project. If a relevant archaic genome has been sequenced, one can map the detected variants to the archaic genome to confirm the source of introgression. We use the genome of the Altai Denisovan and the genome of the Vindija Neanderthal to represent two different sources of archaic introgression. The archaic genomes are in VCF format and the mask files are in BED (Browser Extensible Data) format.

6. For each variant detected by SPrime, map it to the archaic genome, resulting in “match”, “mismatch”, or “notcomp” to the archaic genome. The three states mean the detected variant is present in the archaic genome, is not present in the archaic genome, or is not comparable because genotype quality in the archaic genome is low for that locus. To complete this step, we have an C script named “map_arch”, which adds the match status for each variant as an additional column to SPrime’s output. In the following code, we add match status to the Neanderthal genome and match status to the Denisovan genome.
maparch="../tools/map_arch_genome/map_arch"
for chr in {1..22}; do
  script=o.script.${chr}.sh
  #map variants to the Neanderthal genome
  bedfile="../download/archaic_genome/RecalledVindija/chr${chr}_mask.bed.gz"
  archaicfile="../download/archaic_genome/RecalledVindija/chr${chr}_mq25_mapab100.vcf.gz"
  reftag="AltaiNean"
  scorefile="../step3/chb.yri.${chr}.score"
  outmscore="out.chr${chr}.mscore";
  tmpprefix=../tmp/${RANDOM}
  echo "
  #! /bin/bash
  ${maparch} --kp --sep '\t' --tag ${reftag} --mskbed ${bedfile} --vcf ${archaicfile} --score ${scorefile} > ${tmpprefix}.tmp1.${chr}.mscore
  " >${script}
  #map variants to the Denisovan genome
  bedfile="../download/archaic_genome/RecalledDenisova/chr${chr}_mask.bed.gz"
  archaicfile="../download/archaic_genome/RecalledDenisova/chr${chr}_mq25_mapab100.vcf.gz"
  reftag="AltaiDeni"
  echo "
  ${maparch} –kp –sep '\t' –tag ${reftag} –mskbed ${bedfile} –vcf ${archaicfile} --score ${tmpprefix}.tmp1.${chr}.mscore > ${tmpprefix}.tmp2.${chr}.mscore
  mv ${tmpprefix}.tmp2.${chr}.mscore ${outmscore}
  rm ${tmpprefix}.tmp∗.${chr}.mscore
  rm ${script}
  " >> ${script}
  sh ${script}
done

The mismatch analysis takes 7 mins for chromosome 2 and 81 mins for all autosomes. The maximum memory in use for one chromosome is 10GB.

### Step-5: Find multiple sources of archaic introgression

**Timing: 1 min**

**Figure 2:**
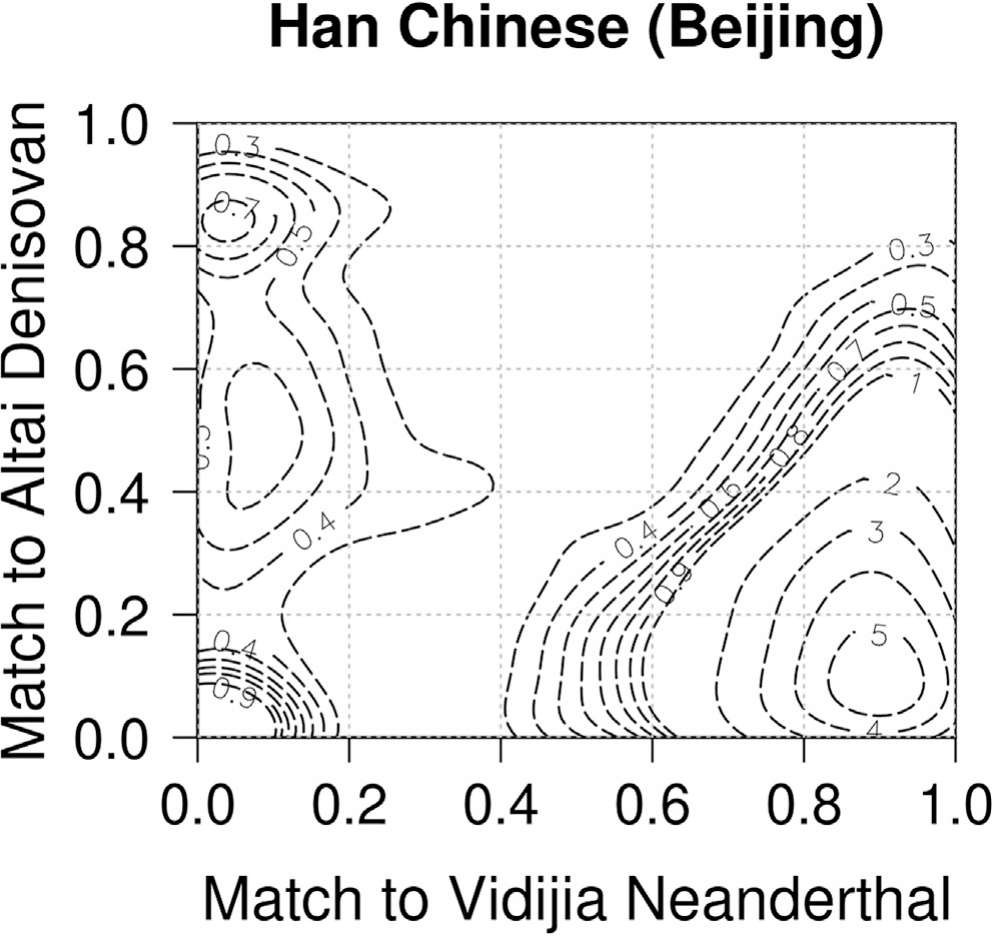
Contour plot of CHB results showing three sources of archaic admixture The peak in the upper left represents ancestry from a group that is closely related to the Altai Denisovan, the peak in the middle left represents ancestry from a group that is distantly related to the Altai Denisovan, the peak on the lower right represents ancestry from Neanderthals, and the peak in the lower left represents other segments which may be false positive introgression calls or introgression from another source.
# calculate match rate for each introgressed segment
## Rscript ../tools/score_summary.r [directory with annotated score files from step 4] [output filename]
Rscript ../tools/score_summary.r ../step4 match.summary.txt
# contour plot show different waves of archaic introgression
## Rscript ../tools/plot_contour.r [input summary file] [prefix of plot output]
Rscript ../tools/plot_contour.r match.summary.txt chb.contour

7. This is an optional step for those who are interested in population history. Once we know the match status of each detected variant to the archaic genome, we are able to calculate the match rate for each reported introgression segment. The match rate for a segment is the number of matching positions divided by the sum of matching and mis-matching positions (the match rate is undefined if all the SPrime variants in the segment are not comparable to the archaic genome). If a segment has high match rate to a particular archaic genome, this segment probably shares close ancestry with that archaic genome. By calculating the match rate to different archaic genomes, we may find evidence of different sources of introgression as in Figure 2 (Browning et al., 2018). The commands to generate this figure are:

## Expected outcomes

**Figure 3:**
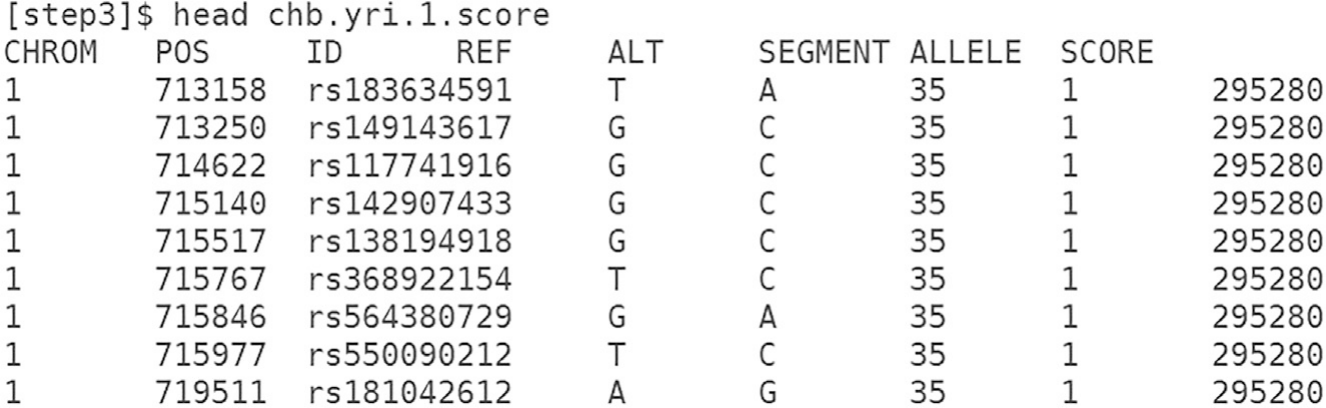
Screenshot of an excerpt of SPrime’s output

SPrimes's output score file from (Figure 3) has 8 columns, including chromosome identifier (CHROM), genome coordinate position (POS), marker identifier (ID), reference allele (REF), alternative allele (ALT), segment index (SEGMENT), introgressed variant (ALLELE) (‘0’ for the reference allele and ‘1’ for the alternative allele), and the score of the introgressed segment (SCORE). SPrime assigns multiple variants to a single segment. Each segment represents a putative introgressed haplotype. The score indicates the confidence of the inference. The higher the score the more likely the segment is introgressed.

**Figure 4:**
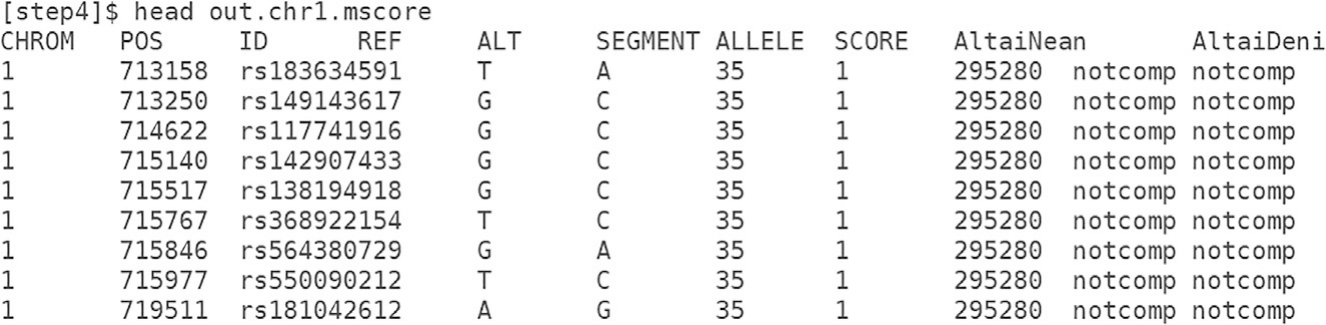
Screenshot of augmented SPrime file with matching to Neanderthal and Denisovan genomes

Comparison of the putative archaic segments to archaic genomes yields one or more additional columns (Figure 4). Although some positions are not represented in the archaic sequences and hence are shown as "notcomp", other positions will "match" or "mismatch" the archaic genome. Within a truly introgressed segment, "match" results will predominate, although some positions will be mismatches due to polymorphism within the archaic population. Visualization of these results is possible through contour plots (Figure 2) for two archaic genomes, or histograms for a single archaic genome.

## Limitations

The SPrime method is based on an out-of-Africa Neanderthal-admixture model, in which a modern human founder population of small size interbred around 60,000 years ago with an archaic population that had split from the main human lineage around 400,000 years ago (Browning et al., 2018). In alternative scenarios, re-tuning of the parameters of the method may be advantageous.

Particular care should be taken when interpreting results from populations in which one or more sub-population split off from the rest of the population a large number of generations ago. If the time since the split has been sufficient for the build-up of a high density of sub-population-specific variants, then admixture with this sub-population can give a signal of archaic introgression in the SPrime results. As an example of such a scenario, the ancestors of the San population in southern African are estimated to have split from other human populations at least 260,000 years ago (Schlebusch et al., 2017).

The SPrime method assumes the availability of sampled individuals from an outgroup population that has experienced negligible admixture from the archaic source. For Neanderthal and Denisovan admixture, West African populations are suitable. However, archaic admixture events that occurred within Africa prior to the out-of-Africa migration may have resulted in current-day admixture in most if not all human populations, so that it may not be possible to find a suitable outgroup population.

The SPrime method doesn't find all the introgressed material because some introgressed segments are too short to be detected. In the out-of-Africa Neanderthal admixture scenario, around one half of introgressed material is detected with the method (Browning et al., 2018). Thus estimates of admixture proportion based on the SPrime results will be underestimates.

## Troubleshooting

### Problem 1

bcftools commands fail (step 2).

### Potential solution

Use “bcftools index” to rebuild the index file for the VCF input.

### Problem 2

SPrime fails (step 3).

### Potential solution

Check the chromosome identifier, which should be consistent between the genotype files and the recombination map file.

### Problem 3

Genotype data is not in GRCh37 (e.g., is in GRCh38), but currently the archaic genomes are only available in GRCh37 (step 4).

### Potential solution

Liftover (see https://genome.ucsc.edu/cgi-bin/hgLiftOver) the SPrime output to GRCh37.

### Problem 4

The “map_arch” program will not run (step 4).

### Potential solution

Confirm that the “zlib” is installed in the system and then type “make” in the folder containing the code to recompile the program.

### Problem 5

The match rate between SPrime's output and the archaic genome is low (steps 4 and 5).

### Potential solutions

The match rate is based on variants that are polymorphic in the target population (such as the CHB) and that are inferred by SPrime to be introgressed from the archaic population. Due to polymorphism in the archaic population, such variants won't always match the archaic genome. The level of divergence between the introgressing population and the population from which the archaic sequence is derived also can also have a significant impact. For example, the match rate between some Denisovan-derived introgressed sequence in the CHB data and the sequenced Altai Denisovan is only 50% due to high divergence, whereas the match rates between Neanderthal-derived introgressed sequence in the CHB and the Vindija Neanderthal is 90% (Figure 2). Genotype errors in the archaic genome, and false positive results in the SPrime analysis also affect results.

False positive rates may be inflated in some populations, such as admixed populations, or when sample sizes are small (<15) (Browning et al., 2018). In such cases, it may be necessary to run simulations to choose an appropriate score threshold.

## Resource availability

### Lead contact

Further information and requests for resources and code should be directed to and will be fulfilled by the lead contact, Sharon Browning (sguy@uw.edu).

### Materials availability

This study did not generate new unique reagents.

### Data and code availability

Code generated during this study is available at https://github.com/YingZhou001/sprimepipeline
